# Supplementary material for: Identification and functional analysis of non-coding regulatory small RNA FenSr3 in Bacillus amyloliquefaciens LPB-18
Source: PeerJ. 2023 May 15;11:e15236. doi: 10.7717/peerj.15236 (PMC10194069; doi:10.7717/peerj.15236)
Supplement: Supplemental Information 4 [file peerj-11-15236-s004.zip › KO/CK-vs-T1_map/map04138.html]

KEGG PATHWAY: Autophagy - yeast - Reference pathway


|  |  |
| --- | --- |
| **Autophagy - yeast - Reference pathway** |  |

[
Pathway menu
| Organism menu
| Pathway entry
| Show description
| User data mapping
]

Autophagy is a non-selective and bulk intracellular degradation system of eukaryotic cells and is highly conserved from yeast to human. In this process, the double-membrane vesicle, known as autophagosome, is formed and sequesters organelles or portions of cytosol. The autophagosome is subsequently fused with the vacuole for breakdown by resident hydrolases, and the resulting metabolites are delivered for reuse. In yeast, nutrient withdrawal is the primary stimulus that induces autophagy. Autophagy plays a central role in normal development and cell homeostasis of yeast.

|  |  |  |
| --- | --- | --- |
| Reference pathway | 184% 150% 122% 100% 82% 67% 55% | 图片下载 |
